# Draft Genome Sequence of Blautia luti DSM 14534^T^, Isolated from Human Stool

**DOI:** 10.1128/MRA.00088-20

**Published:** 2020-05-07

**Authors:** Rodrigo L. Ortiz, Felipe Melis-Arcos, Paulo C. Covarrubias, Juan A. Ugalde, Zachary S. Apte, José Pérez-Donoso, Juan P. Cárdenas, Daniel E. Almonacid

**Affiliations:** a Faculty of Life Sciences, Universidad Andres Bello, Santiago, Chile; b Programa Institucional de Fomento a la Investigación, Desarrollo e Innovación (PIDi), Universidad Tecnológica Metropolitana, Santiago, Chile; c Independent Researcher, Santiago, Chile; d Millennium Initiative for Collaborative Research on Bacterial Resistance (MICROB-R), Santiago, Chile; e Independent Researcher, San Francisco, California, USA; f BioNanotechnology and Microbiology Lab, Center for Bioinformatics and Integrative Biology, Faculty of Life Sciences, Universidad Andres Bello, Santiago, Chile; g Center for Genomics and Bioinformatics, Faculty of Sciences, Universidad Mayor, Huechuraba, Chile; h Escuela de Biotecnología, Faculty of Sciences, Universidad Mayor, Campus Huechuraba, Huechuraba, Chile; i Center for Bioinformatics and Integrative Biology, Faculty of Life Sciences, Universidad Andres Bello, Santiago, Chile; University of Maryland School of Medicine

## Abstract

Here, we report the draft sequence of Blautia luti strain DSM 14534^T^, originally isolated from human feces. This draft contains 74 contigs, comprising 3,718,760 bp with a G+C content of 42.87%. The annotated draft contains 3,338 coding sequences (CDSs) and 110 RNA genes.

## ANNOUNCEMENT

*Blautia*, a genus of anaerobic, nonsporulating, coccobacillus-shaped bacteria from the *Firmicutes* phylum, is a group of Gram-positive bacteria inhabiting the gastrointestinal tract in different animals, including humans (1). Members of this genus are common inhabitants of the healthy human intestinal microbiota (2) and are one of the most abundant groups in adults (3, 4). One member of this genus, Blautia luti (formerly known as Ruminococcus luti), was originally isolated from human feces and characterized as an anaerobic carbohydrate fermenter (5).

The type strain of *B. luti* (DSM 14534) was sequenced from lyophilized pure DNA, directly purchased from the DSMZ repository (catalog number 14534; Germany), using an Illumina MiSeq platform in two independent runs. For each run, a Nextera DNA Flex library prep kit was used to prepare the MiSeq library, and MiSeq paired-end reads were generated using a MiSeq reagent kit v3 (600 cycles; Illumina). Reads from both runs were filtered and trimmed by using Trimmomatic (6), with a minimum quality value (QV) score of <30, a minimum nucleotide length of <100, and removal of Illumina adapters, resulting in 2,200,391 reads (coverage, 173.37×). The filtered reads from both runs were used simultaneously to generate a unique assembly (7) using the SPAdes software v3.13.0 (8) with default parameters, including the additional “-careful” parameter. This resulted in an assembly of 74 contigs, comprising 3,718,760 bp with a G+C content of 42.87%. The *N*_50_ contig size was 205,916 bp, and the longest contig size was 614,844 bp.

The *B. luti* genome completeness and contamination were evaluated using CheckM v1.1.2 (9) with default parameters, showing that the completeness was 99.37% and the contamination was 2.53%. The taxonomic features of the *B. luti* genome confirmed its placement in the *Blautia* genus by use of the “classify_wf” command of GTDB-tk software v0.3.3 with default parameters (10). Subsequently, the assembled genome was annotated with Prokka v1.13 (11) using the “–rfam,” “–gram pos,” and “–genus, –species, –strain” parameters, which resulted in 3,338 coding sequences (CDSs) and 110 RNA genes. Of the CDSs, 1,089 (32.62%) have a cluster of orthologous groups (COG) assigned, and 1,661 (49.76%) correspond to hypothetical proteins. SignalP 5.0b (12) predicted a signal peptide for 326 CDSs (9.77%). Of the RNA genes, 58 were tRNAs, 7 were rRNAs, and 1 was a transfer-messenger RNA (tmRNA) gene. In a dbCAN2 HMM database search with default parameters and the v8 database (13), a total of 91 *B. luti* CDSs (2.7% of the total) were predicted to be glycoside hydrolases (GHs) or glycoside transferases (GTs). This number is within the range of the number of GHs observed in most gut-associated microbes (14).

### Data availability.

The assembled genome sequence of *B. luti* strain DSM 14534^T^ was deposited in GenBank under accession number WMBC00000000. Illumina MiSeq raw reads for the two runs for this sequencing project (BioProject number PRJNA590133) can be accessed under accession numbers SRR10482250 and SRR11245637.
